# Cellular Optimization of Nanofat: Comparison of Two Nanofat Processing Devices in Terms of Cell Count and Viability

**DOI:** 10.1093/asjof/ojz028

**Published:** 2019-09-29

**Authors:** Steven R Cohen, Tunç Tiryaki, Hayley A Womack, Serli Canikyan, Kai Uwe Schlaudraff, Michael Scheflan

**Affiliations:** 1 University of California, San Diego, San Diego, CA; 2 Kansas City University of Medicine and Biosciences, Kansas City, MO; 3 Onkim Stem Cell Technologies, Istanbul Technical University – KOSGEB, Istanbul, Turkey

## Abstract

**Background:**

Nanofat was introduced by Tonnard and Verpaele in 2013. Their initial observations in intradermal applications showed improvement in the appearance of the skin. Since then, a number of Nanofat devices have been introduced. The cellular content in the processing of Nanofat is not the same in every device, yet the cellular composition is responsible for the biologic action of Nanofat. The authors sought to find a different means to produce a matrix rich Nanofat to optimize the cellular content.

**Objectives:**

The primary objective of this study was to compare cell counts, cultures, and cell viabilities produced by LipocubeNano (Lipocube, Inc., London, UK) in comparison to Tulip’s NanoTransfer (Tulip Medical, San Diego, CA) processing methods.

**Methods:**

Twenty milliliters of fat were harvested from 10 patients in order to test two methods of Nanofat production. Ten milliliters of fat were used to assess each method and, after the final product was obtained, enzymatic digestion for stromal vascular fraction (SVF) isolation was performed. A Muse Flow-cytometer was used to measure cell counts and cell viabilities, cell cultures were performed, and cell images were taken with a florescent microscope.

**Results:**

The LipocubeNano was shown to be superior to Tulip’s NanoTransfer system of progressive downsizing with final filtering, which appeared to trap more fibrous tissue leading to lower amounts of SVF. LipocubeNano resulted in higher cell counts (2.24 × 10^6^/cc), whereas Tulip’s NanoTransfer method resulted in a lower cell count at 1.44 × 10^6^/cc. Cell viability was the same (96.05%) in both groups.

**Conclusions:**

Nanofat from LipocubeNano has a higher regenerative cell count and more SVF cells than the other common mechanical method of Nanofat processing. This new means of mechanical processing preserves more matrix, optimizing the cellular content of the Nanofat, thus having potentially a higher regenerative effect.

**Level of Evidence: 5:**

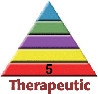

The term “Nanofat” was first conceptualized in 2013 by Tonnard et al when their group mechanically emulsified fat tissue into a liquid form, devoid of connective tissues to be used for the treatment of fine rhytides and other superficial components of facial aging.^1^ Using a 27-gauge needle, Nanofat was injected intradermally into perioral skin, glabellar skin, and breast cleavage, showing improved skin quality. The stromal vascular fraction (SVF) cell count within Nanofat obtained by Tonnard’s group via enzymatic digestion was 1.975 × 10^6^/100 mL lipoaspirate, which demonstrated that his preparation of Nanofat harnessed some SVF cells.^1^ Since its introduction, Nanofat has been utilized for several different regenerative treatments such as scar improvement, burns, radiated tissue, and diabetic wounds.^2–5^ Nanofat is not yet fully defined, but in general, is thought of as fat parcel sizes of 600 microns or less. The cellular composition of Nanofat has been shown to have a lower number of viable adipocytes when compared with that of larger parcel sizes such as macrofat, millifat, and microfat, yet in contrast contains a significant supply of SVF cells that could be injected through smaller gauge needles.^1^

Succeeding the extraction of differentiated adipocytes and removal of debris (RBCs, WBCs, tumescent, etc.), SVF is considered as the residual fibrous extracellular matrix that contains a heterogenous mixture of adipose-derived stem cells, blood cells, pericytes, macrophages, fibroblasts, and vascular endothelial progenitors.^1,6,7^ Numerous studies indicate that SVF cells contribute to the vascularization of autologous fat.^8–11^ Complex adipose tissue that is obtained through the method of entire excision or lipoaspiration conveys mature adipocytes and progenitor cells, which are attached to these adipocytes (preadipocytes are nearly differentiated cells). These progenitor cells symbolize other category of cells having secretory ability in the transferred microenvironment of the fat transfer process. After recognizing the full worth of the complicated, 3-dimensional matrix in adipose tissues, many people think that intact and undistorted transfer of these tissues and other concomitant cellular elements might be advantageous and beneficial for human health.^6,12–14^ The first use of SVF in aesthetics was performed in 2003 by Cohen and Holmes in a series of eight patients. Long-term improvement in wrinkles was observed in one patient 6 years after the study.^15^

In 2015, Aronowitz et al researched several different devices that produced SVF using enzymatic digestion and mechanical isolation techniques.^7^ He pointed out that “not all black boxes are the same” and found variations of cell output and residual collagenase in comparing various methods of SVF isolation. Enzymatic digestion using the Celution system (Cytori, Inc., San Diego, CA) resulted in higher SVF cell counts when compared with other enzymatic separation devices.^7^ In past studies, cell output for enzymatic digestions has yielded much higher SVF cell counts than mechanical isolation.^16,17^ Enzymatic digestion effectively disrupts the collagen-based extracellular matrix binding adipocytes with other cells within adipose tissue, resulting in increased SVF isolation.^7,17^ However, the enzymatic digestion of adipose tissue has been deemed by the FDA as a more than minimal manipulation of tissues, which makes it a clinical disadvantage compared with the minimally manipulative mechanical methods of isolation. 

Currently, there are notable variations in the cell population outputs offered by the mechanical devices available on the market today, which underscores the importance of understanding how different processing options for Nanofat produce their own unique Nanofat products.^18^ Fat grafts removed in larger 1.5- to 2.0-mm parcels are separated into smaller parcel sizes, while preserving as much matrix as possible. Newer mechanical isolation methods have been shown by our group to obtain 25% to 90% of the SVF cell counts that an enzyme produces from the same lipoharvested material; others have validated these findings, although yields have not been reported to be as high as those produced by enzymatic digestion.^7,19–27^

One of the most commonly used mechanical isolation devices for Nanofat production is the Tulip NanoTransfer kit (Tulip Medical, Inc., San Diego, CA), which consists of harvesting through a cannula to obtain generally 1.5- to 2.5-mm fat parcels. The fat parcel size is then reduced using 2.4-, 1.4-, and 1.2-mm connectors that are placed between two syringes (Figure 1). To reach the Nanofat end product, the fat is passed through a device with a two-layered filter of 400 and 600 microns (Figure 2A). We hypothesize these overlapping small-sized filters trap more extracellular matrix, the fibrotic niche of the pericytes, therefore, removing the component where the regenerative SVF cells reside within Nanofat.^7^ However, when the LipocubeNano device is examined, it is observed that the average size of the filter which performs the nano process is 500 microns. Thus, stromal vascular fraction which is under 500 microns stays in suspension which increases total cell count (Figure 2B).

**Figure 1.**
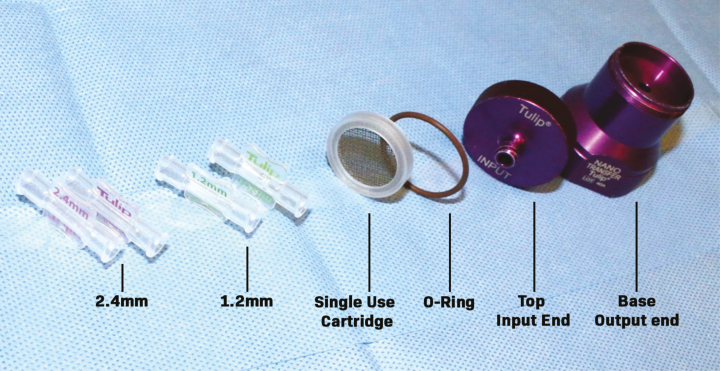
Tulip’s NanoTransfer kit is a single-use closed system which helps size adipose tissue so that it is injectable with 27- and 30-g needles. A proprietary, single-use cartridge is housed in the NanoTransfer.

**Figure 2.**
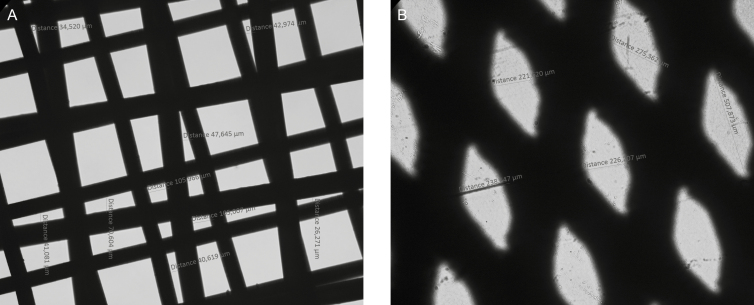
Photographs using light microscopy of (A) NanoTransfer filter vs (B) the LipocubeNano’s cutting screen.

In efforts to optimize SVF cell yields and viability, we designed a “lab in a box,” LipocubeNano, a mechanical isolation device (patent pending, Lipocube Inc, London, UK) designed to maximize the amount of matrix and optimize the cell counts of the Nanofat (Supplementary Figure 1).

Herein, two protocols with two different devices were tested to determine the differences in the cell counts and viability of each of the two Nanofat products: LipocubeNano and the Tulip NanoTransfer Mechanical Isolation Kit. The objectives were to:

1. Determine if the two techniques differed in cell counts and viability and to 2. Determine if by using the principles incorporated into the LipocubeNano, the cellular components in the Nanofat product would be optimized.

## METHODS

This study was conducted in Turkey. The study was conducted under the guiding principles of the Declaration of Helsinki. This study did not require IRB approval as fat-grafting is a long-established procedure and the microinjection device applied in this study received ISO 13485 certification and CE marking. The device is registered and listed with the US Food and Drug Administration (FDA). Patients were preoperatively informed via written consent for all surgical procedures, anesthesia, intraoperative video recording, and photography. Patients consented to donating their adipose tissue for laboratory research and was not clinically investigated. This study was first conducted in February 2019 and concluded after five (5) weeks. Using a 2.4-mm diameter cannula with a 1.8**-**mm hole size under local and tumescent anesthesia, 20 cc’s of lipoaspirate was harvested from 10 consecutive nonobese female patients (*n* = 10) aged between 33 and 54 years (mean age, 47 years) and a mean body mass index of 27 kg/m^2^ (range: 19–34 kg/m^2^). In order to optimize surface area and speed up decanting, the isolations were all cleaned in a small IV bag using warm Ringer’s Lactate Solution. 

### LipocubeNano Mechanical Isolation Method

**Figure 3.**
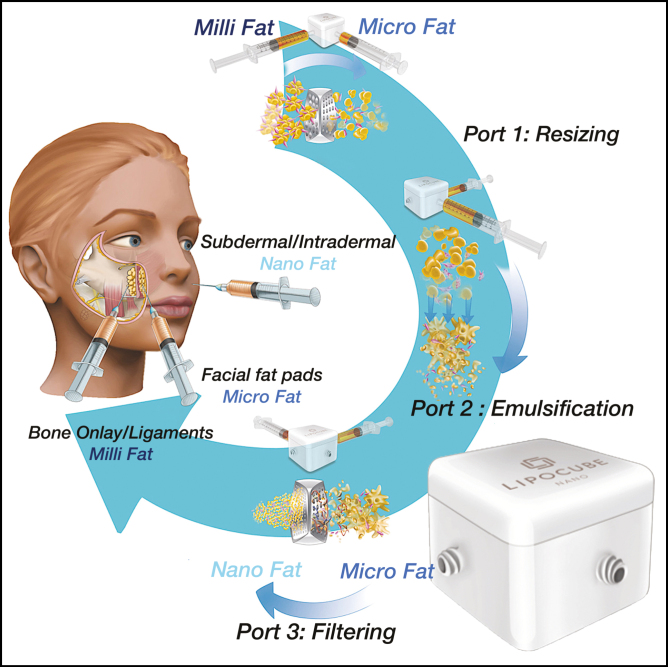
LipocubeNano is a single-use mechanical device for the processing of lipoaspirate, the autologous fat tissue, into milli, micro, and nanofat grafts according to the depth of transfer. The processed fat with LipocubeNano retains the fibrous tissue matrix with regenerative stromal vascular fraction cells.

**Figure 4.**
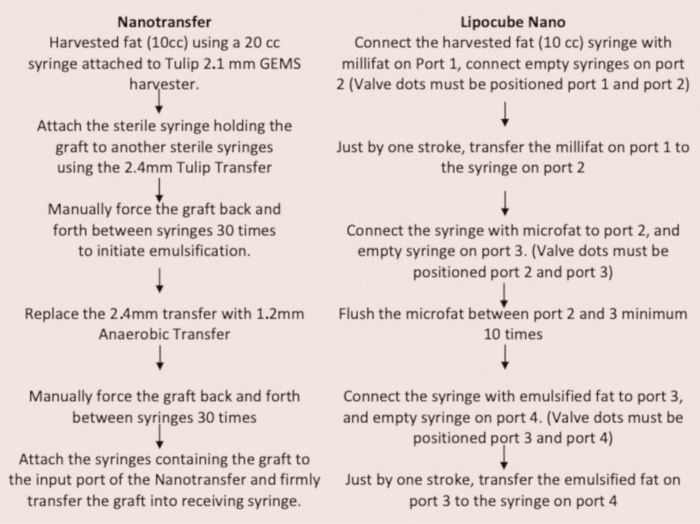
Study methods for fat processing and analysis of contents.

Following cleaning, 10 cc’s of fat were processed with gravity and decanted for 3 min in a syringe. The infranatant fluid was expelled from beneath the graft and processed through the LipocubeNano kit. The fat graft is first passed one time through Port 1, an action which creates 1-mm parcel sizes. The fat is then passed back and forth between Port 2 and Port 3 a total of ten times, which functions to smooth and homogenize the fat tissue. Lastly, the fat was passed one time from Port 3 to Port 4 through a 500-micron single filter to create the Nanofat end product (Figures 3 and 4 and Supplementary Video 1).

### Tulip NanoTransfer Kit Mechanical Isolation Method

Following cleaning, 10 cc’s of fat were processed with gravity and decanted for 3 min in a syringe. The infranatant fluid was expelled from beneath the graft. Using a sterile 2.4-mm Tulip anaerobic transfer, the fat was transferred to a 20 cc holding syringe leaving the supranatant free lipid (clear yellow oil) in the original harvesting syringe. The harvesting syringe was then disposed of. The holding syringe was connected to another sterile 20 cc syringe using a 2.4-mm Tulip transfer and the fat was pushed back forth between the two syringes for a total of 30 times for emulsification. 

In order to size down the fat parcels even farther, the 2.4-mm Tulip transfer was replaced with a 1.2-mm Tulip transfer. The fat was again manually pushed between the two 20 cc syringes for a total of 30 times. Finally, the NanoTransfer device was used to obtain the final Nanofat end-product. The syringe containing the 1.2-mm fat parcels was attached to the input port of the Nanotransfer and the graft was firmly transferred to a receiving syringe through a barrier consisted of two overlapping filters, sized 600 and 400 μm^28^ (Figure 4).

### Nanofat Product Analysis

The end products from both methods were incubated in a proteolytic Collagenase NB6 solution of 0.1U/mL and a ratio of 1:1 (v/v). The digestion was carried out in heated shaker to provide constant agitation at 37°C for 30 min amount of time. The digested adipose tissue was centrifuged at a speed of 300 *g* and duration of 5 min, and the SVF pellets were obtained.

**Figure 5.**
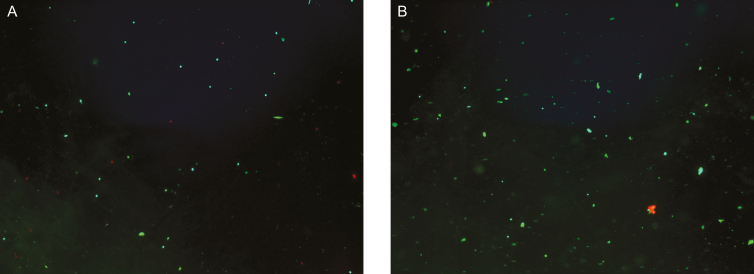
Fluorescent images of (A) NanoTransfer Nanofat and (B) LipocubeNano Nanofat. Note that LipocubeNano has more cell clusters.

Cell counts and cell viabilities were measured using laser-based fluorescence detection via the Muse Flow Cytometer (Merc Millipore, Germany). The Flow Cytometer uses miniaturized fluorescent detection and microcapillary technology to deliver very accurate, precise, quantitative cell analysis compared with other methods. Laser-based fluorescence detection of each cell event can evaluate up to 3 cellular parameters—cell size (forward scatter) and 2 colors (detected in the red and/or yellow channels) (Figure 5).

The characterization of ADSC (CD45−,CD90+/CD73+,CD90+), endothelial cells (CD45−,CD31+), and macrophages and monocytes (CD45+, CD14+) were performed by flow cytometry. The expression of the CD surface markers such as CD13, CD73, CD90, CD146, and CD34 was also examined by flow cytometry. Normal distribution was used for statistics.

Cells were then seeded in T-75 tissue culture plates (Proliferation medium; NutriStem MSC XF Medium/serum free-Biological Industries) at 37°C, at 5% carbon dioxide. After 7 days, cell morphology was observed under light microscopy. We also compared the adipogenic differentiation capacity of ADSC in two groups. Adipogenesis differentiation was carried by StemPro Adipogenesis Differentiation kit according to the manufacturers’ protocol and was evaluated through oil red staining and investigated by phase contrast microscopy.

Gene expression profiles were examined by adipocyte-specific Adiponectin and Ppar genes. Primers were designed using Primer-BLAST software from the National Center for Biotechnology (Bethesda, MD). Total RNA isolation from differentiated cells of two groups was performed according to the manufacturer’s protocol (Total RNA Purification Plus Kit, Norgen, CAN). Student’s *t* test was performed to compare cell count and viability parameters with 95% confidence interval and *P*-values < 0.05.

## RESULTS

The two cell isolation tests were compared using fluorescent staining. The LipoCubeNano produced a cell count of 2.24 × 10^6^/cc with a cell viability of 96.75%. Tulip’s NanoTransfer method resulted at 1.44 × 10^6^/cc with a cell viability of 96.05% (Table 1).

**Table 1.** Cell Number and Cell Viability of Cell (per cc) in the Two Different Groups

	Viable cell number/cc (*n* = 10)	Cell viability (*n* = 10)
Nanofat	1.44 × 10^6^	96.05%
LipocubeNano	2.24 × 10^6^	96.05%

**Figure 6.**
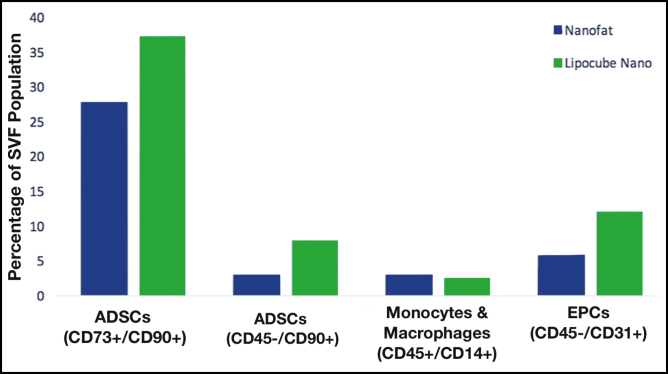
Flow cytometer analysis of SVF subpopulations in the LipocubeNano group vs the NanoTransfer Nanofat group: Endothelial progenitor cells (5.85% vs 11.99%), monocytes macrophages (2.97% vs 2.43%), and ADSCs (27.89% vs 37.29%–3.07% vs 7.92%).

The CD surface markers of fresh ADSC contents in LipocubeNano showed 1.3- and 2.5-fold increase compared with Nanofat (37.29% vs 27.89% and 7.92% vs 3.07%). The endothelial cell content of LipocubeNano was 6.14% higher (11.99% v s 5.85%). The macrophage and monocytes cell content was approximately same as Nanofat (2.43% vs 2.97%) (Figure 6).

**Figure 7.**
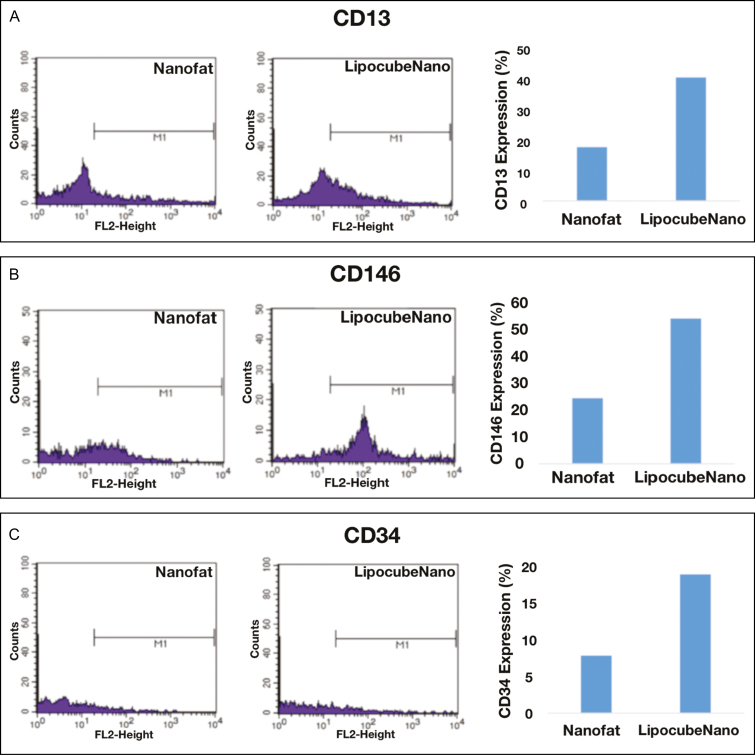

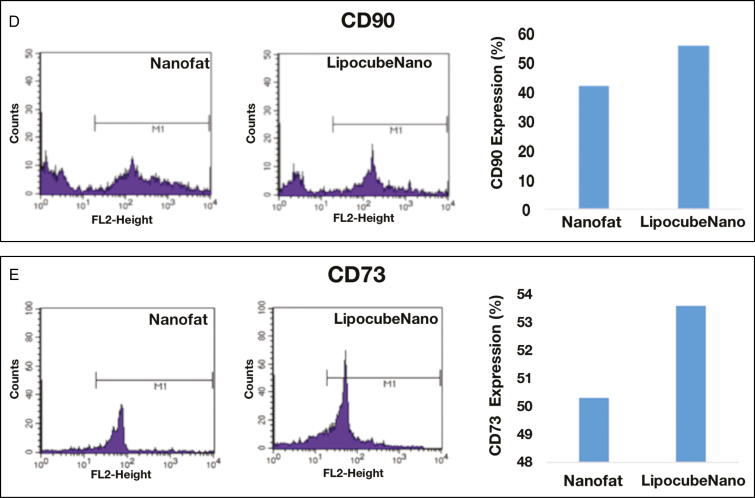
Cell phenotypes by flow cytometer. Comparison between expression of universal stem cell markers. (A) CD13, (B) CD146, (C) CD34, (D) CD90, and (E) CD73 were approximately 1.5-fold higher in the LipocubeNano group in comparison with the Nanofat.

LipocubeNano demonstrated higher expression of specific phenotypic markers. When ADSC markers of cell activity were compared, we observed a 2.3-fold increase in CD13 (42.04% vs 18.28%), a 1.3-fold increase in CD90 (55.82% vs 42.13%), a 2.2-fold increase in CD146 (53.2% vs 24.07%), and a 2.3-fold increase in CD34 (18.84% vs 7.9%) markers which are commonly used stem cell activity markers (Figure 7). CD13 was used instead of CD105 due to its stability as a known ADSC marker.

**Figure 8.**
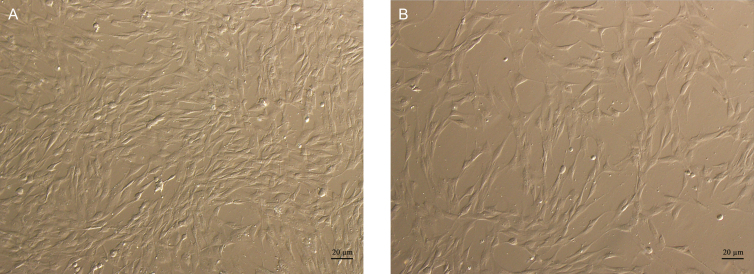
Comparison of differentiation potential via cell culture images of LipocubeNano and NanoTransfer products after 7 days. LipocubeNano Nanofat group had higher adipogenic differentiation examined by phase contrast microscopy at (A) 40× magnification when compared with (B) the NanoTransfer Nanofat group.

After 1 week, cell morphology was observed under light microscopy to compare the adipogenic differentiation capacity of ADSC in LipocubeNano vs NanoTransfer Nanofat groups. LipocubeNano Nanofat group had higher adipogenic differentiation examined by phase contrast microscopy at 40× magnification when compared with the NanoTransfer Nanofat group (Figure 8).

**Figure 9.**
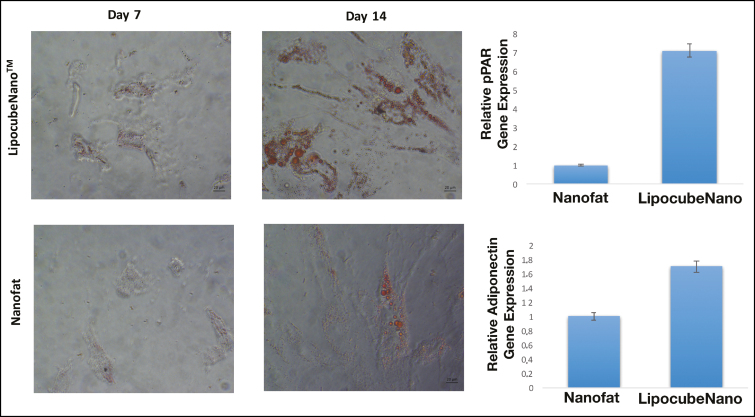
Relative gene expression analysis performed with adiponectin related primers such as PPAR and Adiponectin. 18S was used for the reference housekeeping gene. mRNA levels of the two groups demonstrated that LipocubeNano had approximately 7.1- to 1.7-fold higher PPAR and Adiponectin. Adipogenic differentiation was shown by oil red staining at 7 and 14 days.

Following the differentiation protocol, the mRNA expression levels of PPAR2 and Adiponectin genes were examined. PPAR2 and Adiponectic expression were 7.1- and 1.7-fold higher in LipoCubeNano, respectively, then when compared with NanoTransfer Nanofat group, and showed greater adipogenic differentiation in oil red staining at 7 and 14 days (Figure 9). These findings strongly substantiated that LipoCubeNano process has higher mRNA level of adipocyte complement-related protein which results in a lipid droplets formation, in other words increases adipogenic differentiation by oil red staining (Figure 9).

To date, 32 patients, between ages 40 and 76, with an average age of 56 years, have been treated using a facial fat grafting technique called Injectable Tissue Replacement and Regeneration (ITR^2^) in conjunction with preskeletal fat grafting (pyriform, maxilla, zygoma, mandible) at two centers. The fat harvested from all of these cases were processed using the LipocubeNano. Eleven of these patients had primary facelift surgery, whereas the remaining patients had only ITR^2^ with preskeletal fat grafting. No complications have been reported over a 6-month period since our first treatment. At 6 months, no patients have requested fillers or additional fat grafting treatments. Aesthetic results on a four-point scale have ranged from good to excellent in all patients. 

## DISCUSSION

Nanofat has opened up a number of possibilities in plastic and reconstructive surgery as well as in aesthetic and regenerative medicine. Aesthetic applications for Nanofat include intradermal injection, microneedling as well in combinations with liposomes to make biological cremes that can deliver growth factors and peptides deeper into the skin and possibly into the deeper subcutaneous fat using a transdermal route. The use of Nanofat in combination with laser therapies has been shown to accelerate the rate of healing and improved aesthetic outcomes. Nanofat in combination with a protocol of using multilayer fat grafting techniques such as ITR^2^ and preskeletal fat grafting to anatomically replace lost tissue has shown progressive improvement over a 2-year period when performed with high SMAS facelift surgery.^29^

The several clinical applications available for Nanofat have led to a natural query regarding which processing method can achieve enzyme digestion-comparable SVF cell counts and cell viabilities while remaining under the FDA’s “minimal manipulation” umbrella term. The biologic activity in Nanofat resides in the number of SVF cells and their associated growth factors and cytokines. Cellular optimization techniques are safe, inexpensive, and simple to use.

LipocubeNano is a minimally manipulative device that produces three different types of fat grafts in addition to a matrix and cell enriched Nanofat. In our study, the LipocubeNano was shown to be superior to Tulip’s NanoTransfer system of progressive downsizing with final filtering into a matrix depleted Nanofat. LipocubeNano resulted in a relatively high cell counts (2.24 × 10^6^/cc) and cell viability (96.75%), whereas Tulip’s NanoTransfer method resulted in a lower cell count of 1.44 × 10^6^/cc and a cell viability of 96.75% (Table 1). The results of this study should be reproduced in other laboratory settings and with larger sample sizes to negate any limitations of this study.

In a previous study, Mashiko et al performed a similar comparison of cellular components obtained after fat squeezing technique and an emulsification technique with Tulip’s equipment.^30^ Lipoharvested tissues were centrifuged, centrifuged, and then emulsified further via transfer between two syringes, or squeezed using an automated slicer. Finally, the emulsified tissue was divided, using mesh filtration, into two other portions: residual tissue of emulsified fat and filtrated fluid of emulsified fat. The automated slicer sharply cut the squeezed tissues while the emulsified fat tissues were cut in a blunt manner. Tests such as Immunohistorychemistry, Scanning Electron Microscopy, Flow Cytometry, and SVF Isolation were performed on each of the four products. Mashiko et al’s group reported that the less mechanically manipulated Squeezed fat tissues contained the highest percentage of Extracellular Matrix composition (58.3%), the highest cell count per mL (8 × 10^5^/mL), the lowest percentage of adipocytes, and the highest composition of adipose-derived stromal cells (3.2 × 10^5^) and endothelial cells, with the lowest number of SVF cells in the end stage Nanofat from the Tulip Device.^30^ In addition, the study found the extracellular matrix to have a substantial amount of adipose-derived stromal cells as well as endothelial cells which supports its use in regenerative medicine as found by Feng et al.^5^ Both the fat obtained using LipocubeNano and Mashiko et al’s squeezing method contained higher cell counts than those from Tulip’s fat processing systems. The literature regarding the location of pericytes and adipose derived stem cells, the main workhorse of SVF, suggests that the preservation of the extracellular matrix shall increase the number of regenerative mononuclear cells in the final product, regardless the chosen technique.^31–33^ Our results support the presumption that a less vigorous filtering of the fibrotic tissue generates higher cell numbers of regenerative potential in the cell yield.^31–33^

It is critical that in the field of regenerative medicine, we use similar measurement tools and techniques. Even minor change, for instance, in how long a lysis solution is left in a cell mixture, will influence measurements in different laboratories using the “same technique.” 

The Muse Flow Cytometer was chosen for its precision in discriminating between living and dead cells using fluorescent reagents, which generate signals based on the specificity of unique targets. These fluorescent reagents are chosen based off of compatibility with lasers and detectors and are optimized for multiple cell types. In addition, the Muse Flow Cytometer offers simple “pipette” only assays in which single-cell data can be obtained and statistically analyzed in every sample. 

## CONCLUSION

The LipocubeNano optimized the cell counts and viability of Nanofat, providing a product where the Nanofat cell contents were higher than the current mainstream approaches. Cellular Optimized Nanofat can be obtained through simple, inexpensive devices, and unless a controlled clinical trial showed otherwise, the likelihood of better regenerative outcomes with cell optimized Nanofat makes empiric sense. In addition, using a single device protocol, up to three different fat products can be produced: millifat (parcel sizes of 1.5 to 2 mm), microfat (1 mm), and cell optimized nanofat.

## Disclosures

Dr Cohen has stock options with and receives royalties from Millennium Medical Technologies and receives royalties from Tulip Medical. He is a shareholder in the Mage Group and receives royalties from Lipocube, Ltd. He is an investigator for Allergan, Ampersand, Inc,. and Thermigen. Dr Tiryaki is an investigator for Mentor, receives book royalties from Springer, and is on the advisory board and holds equity in Mage Group and Lipocube Ltd. Mr Canikyan is a genetic engineer at Lipocube Ltd. Drs Schlaudraff and Scheflan are shareholders in Lipocube, Ltd. Ms Womack declared no potential conflicts of interest with respect to the research, authorship, and publication of this article.

## Funding

The authors received no financial support for the research, authorship, and publication of this article.

## Supplementary Material

ojz028_suppl_Supplementary-Figure

ojz028_suppl_Supplementary-Figure-Legend
