# Supplementary figures and images for: Cellular Optimization of Nanofat: Comparison of Two Nanofat Processing Devices in Terms of Cell Count and Viability
Source: Aesthet Surg J Open Forum. 2019 Sep 29;1(4):ojz028. doi: 10.1093/asjof/ojz028 (PMC7780476; doi:10.1093/asjof/ojz028)

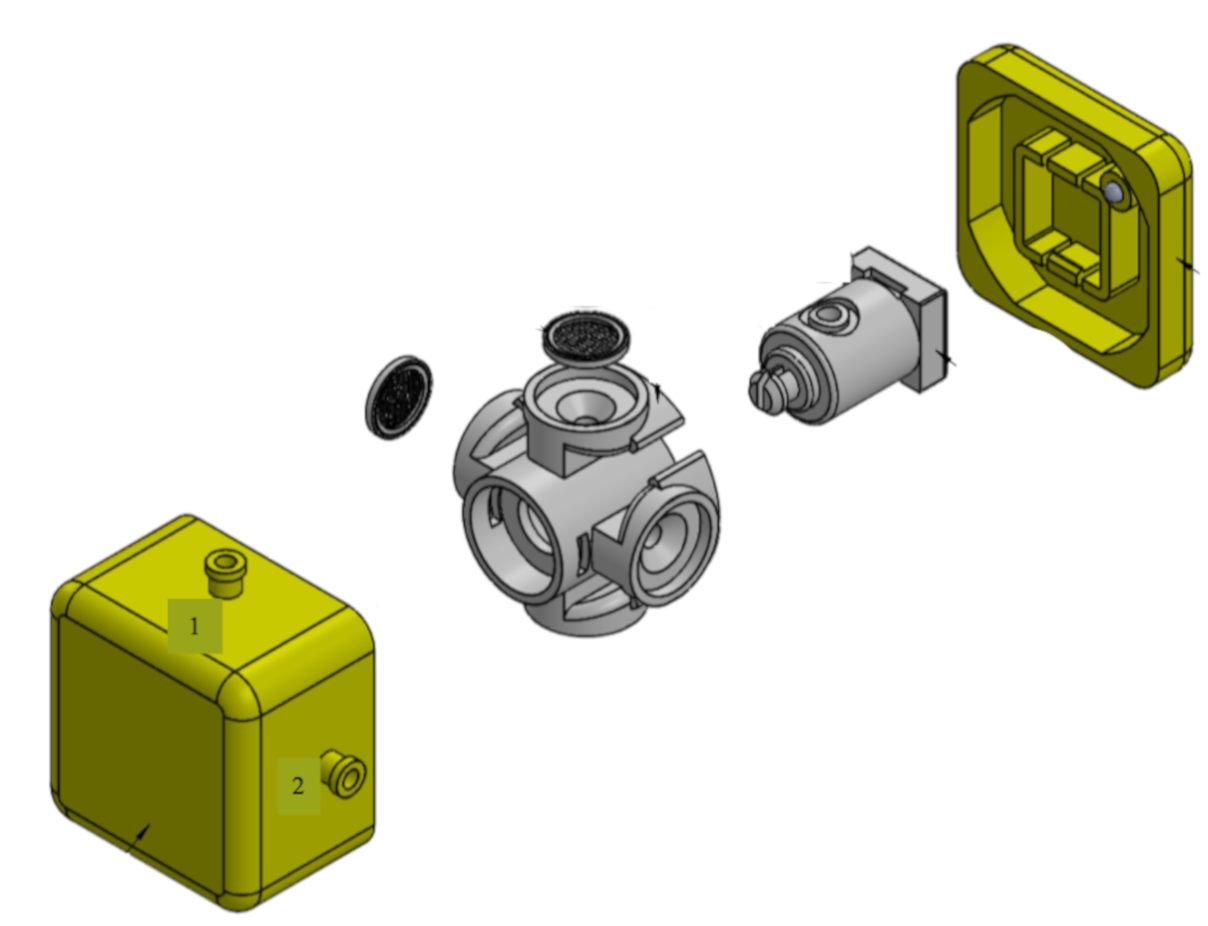

Supplement: ojz028_suppl_Supplementary-Figure [file ojz028_suppl_supplementary-figure.png]
