# Supplementary material for: Cellular Optimization of Nanofat: Comparison of Two Nanofat Processing Devices in Terms of Cell Count and Viability
Source: Aesthet Surg J Open Forum. 2019 Sep 29;1(4):ojz028. doi: 10.1093/asjof/ojz028 (PMC7780476; doi:10.1093/asjof/ojz028)
Supplement: ojz028_suppl_Supplementary-Figure-Legend [file ojz028_suppl_supplementary-figure-legend.docx]

**Supplemental Figure 1.** A technical illustration of the LipocubeNano^TM^ device. In this diagram, every individual part of the device can be observed.
